# The Effect of *APOE ε*4 on Alzheimer's Disease Fluid Biomarkers: A Cross‐Sectional Study Based on the COAST

**DOI:** 10.1111/cns.70202

**Published:** 2025-01-03

**Authors:** Bote Zhao, Peixi Zang, Meina Quan, Qianqian Wang, Dongmei Guo, Jianping Jia, Wei Wang

**Affiliations:** ^1^ Innovation Center for Neurological Disorders and Department of Neurology, Xuanwu Hospital Capital Medical University Beijing China; ^2^ National Center for Neurological Disorders and National Clinical Research Center for Geriatric Diseases Beijing China; ^3^ Center of Alzheimer's Disease, Beijing Institute of Brain Disorders Collaborative Innovation Center for Brain Disorders Beijing China; ^4^ Department of Neurology Gansu Provincial Hospital Lanzhou City Gansu Province China

**Keywords:** Alzheimer's disease, *apolipoprotein* E, biomarker, high‐density lipoprotein, immunoglobulin, low‐density lipoprotein, uric acid

## Abstract

**Aims:**

To analyze the effect of *APOE ε*4 on fluid biomarkers and the correlations between blood molecules and CSF biomarkers in AD patients.

**Methods:**

This study enrolled 575 AD patients, 131 patients with non‐AD dementia, and 112 cognitively normal (CN) participants, and AD patients were divided into *APOE ε*4 carriers and non‐carriers. Cerebrospinal fluid (CSF) biomarkers and blood‐derived biomolecules were compared between AD and CN groups, between non‐AD dementia and CN groups, as well as within *APOE ε*4 subgroups of AD patients. Utilizing Spearman's correlation analysis and quantile regression analysis, the relationships between blood‐derived biomolecules and CSF biomarkers were analyzed in *APOE ε*4 carriers and non‐carriers.

**Results:**

The levels of CSF biomarkers and blood molecules exhibited significant differences between the AD and CN groups, including Aβ42, t‐tau, p‐tau 181, high‐density lipoprotein, low‐density lipoprotein (LDL), and uric acid. In AD patients, *APOE ε*4 carriers had increased levels of CSF t‐tau, p‐tau 181, and plasma LDL. In the correlation and regression analyses, the negative relationships between plasma TG and t‐tau, between plasma TG and p‐tau 181 levels, as well as the positive relationship between serum IgA and CSF Aβ42, were observed significantly in *APOE ε*4+ AD groups, but not in *APOE ε*4− AD group.

**Conclusion:**

*APOE ε*4 is associated with accelerated progression of AD pathology. The blood‐derived biomolecules correlated with CSF biomarkers in *APOE ε*4 carriers are related to neuroinflammation and lipid metabolism, which may indicate the role of *APOE ε*4 in AD pathophysiology and offer insights for diagnostic and therapeutic strategies for AD.

**Trial Registration:**

ClinicalTrials.gov identifier: NCT03653156

## Graphical abstract

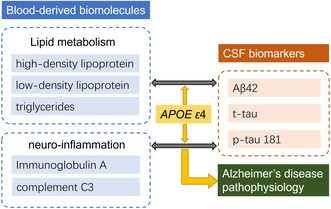

## 1 Introduction

Alzheimer's disease (AD) stands as one of the most challenging and complex neurodegenerative disorders nowadays, affecting millions of individuals worldwide [1]. The *apolipoprotein E* (*APOE*) gene has garnered considerable attention for its association with risk of AD [2, 3]. The *APOE* gene is characterized by three common alleles, known as *ε*2, *ε*3, and *ε*4. Each individual inherits one allele from each parent, leading to the formation of six distinct genotypic combinations. Notably, the *ε*4 allele has been consistently associated with an increased risk of developing sporadic AD [4, 5], while the precise mechanisms through which *APOE ε*4 contributes to AD pathology are complex and not fully understood.

Recent research has turned its focus toward discovering the influence of *APOE ε*4 on AD biomarkers, which include amyloid‐beta (Aβ) and tau proteins in cerebrospinal fluid (CSF), as well as neuroimaging markers [4, 6]. The relationships between *APOE ε*4, CSF, and blood biomarkers have been discussed in several studies, which indicate that *APOE* genotypes may have effects on several CSF biomarkers and blood‐derived biomolecules, including cholesterol, immunoglobulin, complement factor, uric acid (UA), etc [7, 8, 9, 10, 11, 12, 13]. However, the relationships between *APOE ε*4 and blood‐derived biomolecules, as well as the correlations between CSF biomarkers and blood‐derived biomolecules, are still in need of further investigation.

Moreover, blood‐based biomarkers are less medically invasive and less expensive, when compared to CSF and PET biomarker assessments. Thus, blood‐based biomarker measurements have been recognized as a valuable method for early diagnosis and monitoring of AD progression [14]. The identification of novel blood‐based biomarkers for AD is imperative, and exploring the correlations between blood‐derived biomolecules and CSF biomarkers may facilitate the discovery of potential blood biomarkers for AD.

Based on cross‐sectional data from an ongoing cohort study, we analyzed the effects of *APOE ε*4 on AD biomarkers in CSF and blood‐derived biomolecules. Additionally, we investigated the correlations between CSF biomarkers and blood‐derived biomolecules in AD patients stratified according to *APOE ε*4 carrying status, aiming to provide a comprehensive understanding of how *APOE* genotypes might influence AD pathophysiology through the blood‐derived biomolecules. This study could illuminate the associations between *APOE* genotypes, AD biomarkers, and blood‐derived biomolecules, thus providing valuable insights that may pave the way for novel diagnostic and therapeutic strategies for AD.

## 2 Methods

### 2.1 Study Design and Participants

**FIGURE 1:**
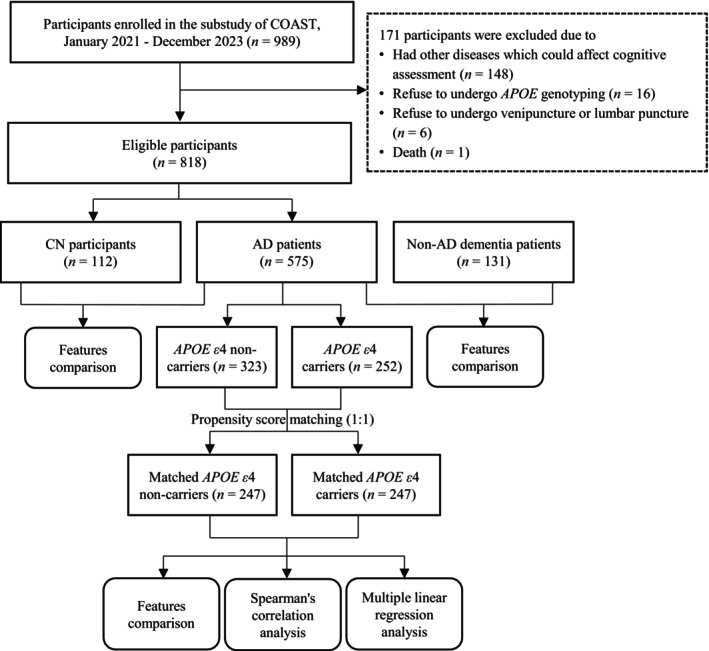
Study design. The patients with non‐AD dementia included patients with frontotemporal dementia, Parkinson's Disease dementia, and Lewy Body dementia. The other diseases which could affect cognitive assessment included corticobasal degeneration, multiple sclerosis, substance abuse, severe systematic diseases, active infections, chronic wasting disease, autoimmune diseases, hematological system diseases, newly onset cardiovascular diseases, stroke, and traumatic brain injury. In AD patients, the *APOE ε*4 carriers were matched with the non‐carriers by age, sex, and educational level. AD, Alzheimer's disease; *APOE*, apolipoprotein E; CN, cognitively normal; COAST, China Cognition and Aging Study.

The China Cognition and Aging Study (COAST) is a nationwide prospective cohort study on dementia in China (Study ID Number: SYXWJ001). In this cross‐sectional study based on the COAST, we recruited AD patients who met the enrollment criteria from January 2021 to December 2023. Patients diagnosed with AD, according to the National Institute of Aging and Alzheimer's Association (NIA‐AA) criteria [15], were consecutively enrolled from the Innovation Center for Neurological Disorders, Department of Neurology, Xuanwu Hospital, Capital Medical University. A total of 112 cognitively normal (CN) individuals were identified from subjects who had undergone routine medical assessment in the hospital. Another 131 patients with non‐AD dementia (including frontotemporal dementia, Parkinson's disease dementia, and Lewy Body dementia) were also enrolled from the inpatients of Xuanwu Hospital, Capital Medical University. The CN participants and the patients with non‐AD dementia (non‐ADD) were age‐matched with the AD patients (Figure 1).

The protocol of this study was approved by the Ethics Committee of Xuanwu Hospital, Capital Medical University, and followed the tenets of the Helsinki Declaration. Written or oral informed consent was obtained from the participants or their legal guardians prior to the study procedures.

In this study, we defined the inclusion criteria as participants who (1) had complete basic demographic data (including age and sex); (2) underwent both one blood sample assessment and one CSF sample assessment from January 2021 to December 2023; (3) underwent *APOE* genotyping at baseline. The exclusion criteria of this study were defined as participants who (1) had corticobasal degeneration, multiple sclerosis, or substance abuse; (2) had severe systematic diseases, active infections, chronic wasting disease, autoimmune diseases, hematological system diseases, or cancer; (3) had newly onset cardiovascular diseases or stroke; (4) had traumatic brain injury recently; (5) refuse to undergo venipuncture for blood sample assessment or lumbar puncture for CSF collection; (6) refuse to sign the informed consent.

### 2.2 Collection of Demographic Information

All of the eligible participants underwent collection of demographic information, including age, sex, education level, body mass index (BMI), history of drinking, smoking, hypertension, hyperlipidemia, and diabetes mellitus. The following information was self‐reported by the participants or the legal guardians of the patients, including education level, history of drinking, smoking, hypertension, hyperlipidemia, and diabetes mellitus.

### 2.3 Assessments of Cognitive Function

The global cognitive function of the participants was assessed by the Mini‐Mental State Examination (MMSE) and the Montreal Cognitive Assessment (MoCA) scales [16, 17]. Besides meeting other inclusion and exclusion criteria, the MMSE scores of the cognitively normal group should also fall within the normal range: MMSE > 22 points for illiteracy, > 23 points for those with primary school education, > 24 points for those with middle school education, > 26 points for those with college education or above [16, 18, 19]. The neurology physician who performed the cognitive test and gave AD diagnosis to the patients was blinded to the results of blood, CSF sample, and *APOE* alleles testing.

### 2.4 Blood Sample Assessment

All individuals had undergone routine blood testing. Fasting plasma samples were collected before 8 a.m. by venipuncture in heparin‐lithium anticoagulant tubes. After centrifugation, serum was then separated and stored at −80°C until assayed. Utilizing standard assays, the plasma samples were divided into aliquots and placed in polypropylene tubes for biochemical analyses. In this study, we focused on four variables in the biochemical blood test, including UA, high‐density lipoprotein (HDL), low‐density lipoprotein (LDL), and triglycerides (TG). Nonfasting blood samples were collected with 5 mL serum‐separating vacutainer tubes and centrifuged at 1300 *g* for 10 min to separate the serum for immunological test. The serum‐derived molecules which underwent further analysis included Immunoglobulin A (IgA) and complement 3 (C3).

### 2.5 CSF Sample Assessment

Samples of CSF from all the eligible participants were obtained by lumbar puncture in the L3/4 or L4/5 interspace without reported serious adverse effects, collected in polypropylene tubes, centrifuged, and stored frozen at −80°C until analysis, according to standard operating procedures. The concentrations of the biomarkers in CSF were measured with the use of enzyme‐linked immunosorbent assay kits according to the manufacturers' instructions. The kits used for the measurement of AD biomarkers in CSF were INNOTEST β‐AMYLOID (1–40) for Aβ40, INNOTEST β‐AMYLOID (1–42) for Aβ42, INNOTEST hTAU Ag for total tau protein (t‐tau), and INNOTEST PHOSPHO‐TAU (181P) for phosphorylated tau 181 (p‐tau 181), all FUJIREBIO. All biomarker results were required to adhere to the quality‐control standards, which included ensuring that biomarker concentrations fell within the specified assay ranges of the respective kits and maintaining consistent measurement uniformity across all plates by using a validation control included on each plate. Furthermore, to protect the confidentiality of the participants and to minimize the selection bias, the laboratory technicians who conducted the CSF sample analyses were blinded to the diagnosis and other clinical information related to this study of the participants.

### 2.6 *APOE* Alleles Testing

Genotyping for *APOE* (gene map locus 19q13.2) was conducted utilizing allelic discrimination technology (TaqMan; Applied Biosystems) or equivalent methods. Genotypes for the two single‐nucleotide polymorphisms (rs429358 C/T and rs7412 C/T), which define *APOE ε*2, *ε*3, and *ε*4, were determined using real‐time fluorescence quantitative polymerase chain reaction with nucleic acid detection reagents. The *APOE ε*4 carriers (*APOE ε*4+ group) were defined as participants who carried the *APOE ε*4/ *ε*4, *APOE ε*3/ *ε*4, or *APOE ε*2/ *ε*4, while the *APOE ε*4 non‐carriers (*APOE ε*4− group) were defined as the ones who carried the *APOE ε*2/ *ε*2, *APOE ε*2/ *ε*3 or *APOE ε*3/ *ε*3.

### 2.7 Statistical Analysis

For the continuous numerical variables, outliers of each variable were defined as with z‐scores over three and were excluded from the following analysis [20]. In AD patients, propensity score matching (PSM) was conducted to match the *APOE ε*4 carriers with the non‐carriers by age, sex, and education level. Demographic variables, cognitive function, the levels of AD biomarkers in CSF, and the blood‐derived biomolecules between the AD patients and the CN participants, between the patients with non‐AD dementia and the CN participants, as well as between the matched *APOE ε*4 carriers and non‐carriers of AD patients were compared. Kolmogorov–Smirnov test was used to test for normal distribution. Continuous variables conforming to normal distribution were presented as means (SD) and compared by a two‐tailed *t*‐test, while non‐normal distributed continuous variables were presented as median (interquartile range) and compared by nonparametric test. Categorical variables were presented as number (percentage) and compared by Chi‐Squared test (*χ* ^2^ test). Statistical significance was defined as a two‐sided *p* < 0.05. The correlations of blood‐derived biomolecules and AD biomarkers in CSF were assessed using Spearman's correlation analysis and visually represented in heat maps. Quantile regression analyses were further conducted to validate the differences of correlations between blood‐derived biomolecules and CSF biomarkers in *APOE ε*4 subgroups. The models were all constructed at 0.50 quantile and adjusted by confounding factors including age, sex, education level, and disease duration. To account for multiple testing, two‐sided *p*‐values were adjusted according to the method of Benjamini/Hochberg (B/H) to control the false discovery rate. An association was considered to be statistically significant, if its corresponding B/H‐adjusted *p*‐value was below 0.05, corresponding to an FDR of 5%. Statistical analyses were conducted using the R software, version 4.3.2 (R Foundation for Statistical Computing, Vienna, Austria). We used the following R packages: “*MatchIt*” for PSM; “*corrplot*” and “*ggplot2*” for plotting of correlation heatmaps; “*quantreg*” for quantile regression analysis.

## 3 Results

In total, 989 individuals were screened for eligibility, during which 148 patients with other diseases which could affect cognitive assessment and one dead patient were excluded. Participants who refused to undergo *APOE* genotyping (*n* = 16) or venipuncture and lumbar puncture (*n* = 6) were also excluded. A total of 818 individuals (575 AD patients, 131 patients with non‐AD dementia, and 112 CN participants) aged between 50 and 87 years old were eligible for this study (Figure 1). The median age was 64 years old, and 457 (55.9%) participants were females. Of all the participants included in this study, 342 (41.8%) cases carried *APOE ε*4 allele. Among the 575 AD patients enrolled in this study, 252 cases (43.8%) were *APOE ε*4 carriers (Table 1). The number and the proportion of missing data and outliers (with z‐scores over three) for each variable used for further analysis are shown in Table S1.

**TABLE 1:** Demographic and clinical features of CN participants, AD and non‐ADD patients.

Characteristics	CN	AD	Non‐ADD	*p* (AD vs. CN)	*p* (non‐AD vs. CN)
Participants, *n*	112	575	131		
Female, *n* (%)	58 (51.8)	344 (59.8)	76 (58.0)	0.162	0.162
Age (years), median (IQR)	64.50 (60.75, 68.25)	64.00 (58.00, 70.00)	64.00 (59.00, 69.00)	0.916	0.916
*APOE ε*4 carriers, *n* (%)	41 (36.6)	252 (43.8)	49 (37.4)	0.382	0.999
Education level, *n* (%)					
Illiteracy	0 (0.0)	20 (3.7)	2 (1.8)	0.029*	0.098
Primary school	9 (8.5)	75 (13.9)	19 (17.0)		
Junior high school	26 (24.5)	133 (24.7)	24 (21.4)		
Senior high school	30 (28.3)	152 (28.3)	40 (35.7)		
Bachelor's degree	39 (36.8)	146 (27.1)	27 (24.1)		
Master's degree and above	2 (1.9)	12 (2.3)	0 (0.0)		
BMI (kg/m^2^), median (IQR)	24.22 (22.49, 27.09)	23.44 (21.39, 25.35)	23.81 (22.03, 25.77)	0.002**	0.254
Smoking, *n* (%)	24 (21.4)	116 (20.2)	36 (27.5)	0.862	0.694
Drinking, *n* (%)	24 (21.4)	99 (17.2)	36 (27.5)	0.353	0.353
History					
Hypertension, *n* (%)	47 (42.0)	197 (34.4)	51 (38.9)	0.308	0.727
Diabetes mellitus, *n* (%)	21 (18.8)	100 (17.5)	20 (15.3)	0.852	0.852
Hyperlipidemia, *n* (%)	15 (13.4)	60 (10.4)	14 (16.5)	0.689	0.689
Cognitive function					
MMSE, median (IQR)	27 (25, 28)	18 (12, 23)	19 (13, 23)	< 0.001***	< 0.001***
MoCA, median (IQR)	22 (20, 25)	13 (8, 18)	12 (7, 17)	< 0.001***	< 0.001***

*Note:* Data were presented as number (percentage), mean ± SD, or median (IQR). All *p* values were adjusted by Benjamini/Hochberg (B/H) method.

Abbreviations: AD, Alzheimer's disease; BMI, body mass index; CN, cognitively normal; IQR, interquartile range; MMSE, mini‐mental State Examination; MoCA, Montreal Cognitive Assessment; non‐ADD, non‐AD dementia; SD, standard deviation.

* B/H adjusted *p* < 0.05.

** B/H adjusted *p* < 0.01.

*** B/H adjusted *p* < 0.001.

### 3.1 Differences of Clinical Features in CN, AD, and Non‐ADD Groups

**FIGURE 2:**
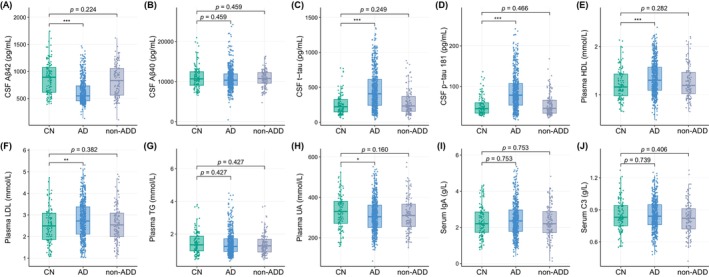
Differences of CSF biomarkers and blood biomolecules between the groups of CN, AD, and non‐ADD. All *p* values were adjusted by Benjamini/Hochberg (B/H) method. Aβ, amyloid‐beta; AD, Alzheimer's disease; CN, cognitively normal; CSF, cerebrospinal fluid; C3, complement C3; HDL, high‐density lipoprotein; IgA, Immunoglobulin A; LDL, low‐density lipoprotein; non‐ADD, non‐AD dementia; p‐tau, phosphorylated tau; TG, triglycerides; t‐tau, total tau; UA, uric acid. *B/H adjusted *p* < 0.05, **B/H adjusted *p* < 0.01, ***B/H adjusted *p* < 0.01.

The demographic characteristics and levels of CSF biomarkers and blood molecules from the groups of CN, AD, and non‐ADD are exhibited in Table 1 and Figure 2. AD patients were more likely to have a lower BMI, when compared with CN participants (*p* = 0.001). AD patients demonstrated significantly lower scores in cognitive assessments (MMSE and MoCA) (*p* < 0.001) in comparison to the CN group (Table 1). The levels of CSF Aβ42 and plasma UA from the AD patients were significantly lower than that from the CN group (*p* < 0.001; *p* = 0.016). The levels of CSF t‐tau and p‐tau 181 levels in CSF, as well as plasma HDL and LDL from the AD patients were significantly higher than that from the CN group (*p* < 0.001; *p* < 0.001; *p* < 0.001; *p* = 0.004) (Table 1, Figure 2). However, patients with non‐AD dementia only demonstrated significantly lower scores in cognitive assessments (MMSE and MoCA) (*p* < 0.001) in comparison to the CN group. The levels of CSF biomarkers and blood‐derived molecules did not exhibit differences with statistical significance between CN and non‐ADD groups (Table 1).

### 3.2 Differences of Clinical Features in *APOE ε*4+ and *APOE ε*4− AD Patients

**FIGURE 3:**
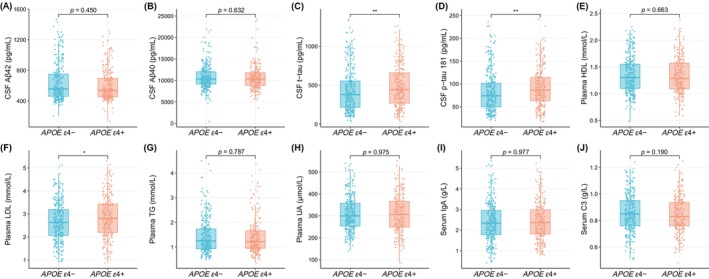
Differences of CSF biomarkers and blood biomolecules between the *APOE ε*4‐ and *APOE ε*4+ groups in AD patients. Aβ, amyloid‐beta; AD, Alzheimer's disease; *APOE*, *apolipoprotein E*; CSF, cerebrospinal fluid; C3, complement C3; HDL, high‐density lipoprotein; IgA, Immunoglobulin A; LDL, low‐density lipoprotein; p‐tau, phosphorylated tau; TG, triglycerides; t‐tau, total tau; UA, uric acid. *p < 0.05, **p < 0.01.

In AD patients, 247 *APOE ε*4 non‐carriers were successfully matched with *APOE ε*4 carriers in a ratio of 1:1, by PSM adjusted for age, sex, and education level. In Table 2, we summarized the demographic characteristics and levels of fluid biomarkers from the matched *APOE ε*4 carriers and non‐carriers. Demographic information, including age, sex, AOO, disease duration, educational level, smoking, drinking, BMI, etc., demonstrated no significant difference between the two *APOE* subgroups. The results of cognitive function assessments (including MMSE and MoCA), and levels of some blood‐derived biomolecules (including HDL, TG, UA, IgA, and C3) indicated no significant difference between the two groups (Table 2). The levels of plasma LDL, CSF t‐tau, and p‐tau 181 from the *APOE ε*4 carrier group were significantly higher than that from the non‐carrier group in AD patients (*p* = 0.015, *p* = 0.001, *p* = 0.001) (Table 2, Figure 3).

**TABLE 2:** Demographic and clinical features of matched *APOE ε*4− and *APOE ε*4+ AD patients.

Characteristics	All matched AD patients	Matched *APOE ε*4− AD patients	Matched *APOE ε*4+ AD patients	*p*
Patients, *n*	494	247	247	
Female, *n* (%)	307 (62.1)	151 (61.1)	156 (63.2)	0.711
Age (years), median (IQR)	65 (58, 70)	65 (58, 70)	66 (59, 71)	0.490
AOO (years), median (IQR)	62 (55, 68)	62 (55, 67)	63 (55, 68)	0.511
Disease duration (years), median (IQR)	2 (1, 4)	2 (1, 3)	2 (1, 4)	0.341
Education level, *n* (%)				
Illiteracy	17 (3.7)	9 (3.9)	8 (3.5)	0.267
Primary school	67 (14.5)	32 (13.7)	35 (15.2)	
Junior high school	118 (25.5)	51 (21.9)	67 (29.1)	
Senior high school	125 (27.0)	70 (30.0)	55 (23.9)	
Bachelor's degree	125 (27.0)	63 (27.0)	62 (27.0)	
Master's degree and above	11 (2.3)	8 (3.5)	3 (1.3)	
BMI (kg/m^2^), mean ± SD	23.52 ± 3.14	23.46 ± 2.96	23.57 ± 3.31	0.692
Smoking, *n* (%)	95 (19.2)	48 (19.4)	47 (19.0)	0.999
Drinking, *n* (%)	81 (16.4)	43 (17.4)	38 (15.4)	0.627
History				
Hypertension, *n* (%)	167 (33.9)	78 (31.6)	89 (36.3)	0.309
Diabetes mellitus, *n* (%)	88 (17.9)	50 (20.2)	38 (15.6)	0.218
Hyperlipidemia, *n* (%)	54 (10.9)	33 (13.4)	21 (8.5)	0.113
Cognitive function				
MMSE, median (IQR)	18 (12, 23)	18 (12, 24)	19 (13, 23)	0.698
MoCA, median (IQR)	13 (8, 18)	13 (8, 19)	13 (8, 18)	0.904

*Note:* Data were presented as number (percentage), mean ± SD, or median (IQR). *APOE ε*4−, *APOE ε*4 non ‐ carriers; *APOE ε*4+, *APOE ε*4 carriers.

Abbreviations: AD, Alzheimer's disease; AOO, age of onset; *APOE*, *apolipoprotein E*; BMI, body mass index; IQR, interquartile range; MMSE, Mini‐mental State Examination; MoCA, Montreal Cognitive Assessment; SD, standard deviation.

### 3.3 Correlation Analysis

**FIGURE 4:**
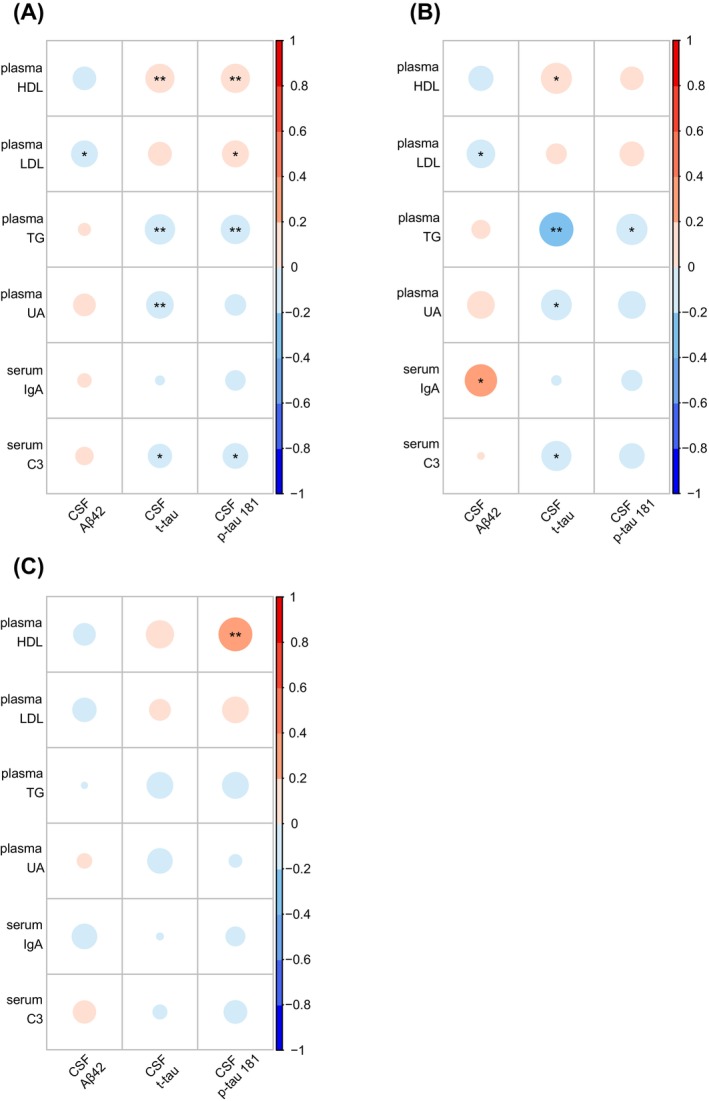
Heat maps of correlations between blood‐derived biomolecules and CSF biomarkers. Shown are the correlations between blood‐derived biomolecules and CSF biomarkers in all matched AD patients (A), *APOE ε*4+ AD patients (B), and *APOE ε*4− AD patients (C). All *p* values were adjusted by Benjamini/Hochberg (B/H) method. Aβ, amyloid‐beta; AD, Alzheimer's disease; *APOE*, *apolipoprotein E*; CSF, cerebrospinal fluid; C3, complement C3; HDL, high‐density lipoprotein; IgA, Immunoglobulin A; LDL, low‐density lipoprotein; p‐tau, phosphorylated tau; TG, triglycerides; t‐tau, total tau; UA, uric acid. *B/H adjusted *p* < 0.05, **B/H adjusted *p* < 0.01, ***B/H adjusted *p* < 0.001.

Among the 494 AD patients selected by PSM, correlations between the blood‐derived biomolecules with AD biomarkers in CSF are presented in Figure 4 and Table S2, as are the results from the *APOE ε*4 subgroups (Figure 4; Table S3). The negative correlations between plasma LDL and CSF Aβ42 levels (*r* range: −0.163 to −0.109), between plasma TG and CSF t‐tau levels (*r* range: −0.235 to −0.184), between plasma UA and CSF t‐tau levels (*r* range: −0.190 to −0.152), between serum C3 and CSF t‐tau levels (*r* range: −0.182 to −0.116), and between plasma TG and CSF p‐tau 181 levels (*r* range: −0.195 to −0.172), as well as the positive correlation between plasma HDL and CSF t‐tau levels (*r* range: 0.169 to 0.193) were observed significant (B/H adjusted *p* < 0.05) in total AD and *APOE ε*4+ AD groups, but not in *APOE ε*4− AD group (Figure 4; Table S2, 3). The level of serum IgA was positively and significantly correlated with CSF Aβ42 level (*r* = 0.211, B/H adjusted *p* = 0.030) only in *APOE ε*4+ AD group (Figure 4; Table S3). The level of plasma HDL was positively and significantly correlated with CSF p‐tau 181 level in total AD and *APOE ε*4− AD groups (*r* range: 0.169 to 0.231, B/H adjusted *p* < 0.05), but not in *APOE ε*4+ AD group (Figure 4; Table S2, 3). The positive correlation between plasma LDL and CSF p‐tau 181 levels (*r* = 0.143), as well as the negative correlation between serum C3 and CSF p‐tau 181 levels (*r* = −0.130) were observed significant (B/H adjusted *p* < 0.05) only in total AD group (Figure 4; Table S2).

### 3.4 Quantile Regression Analysis

**FIGURE 5:**
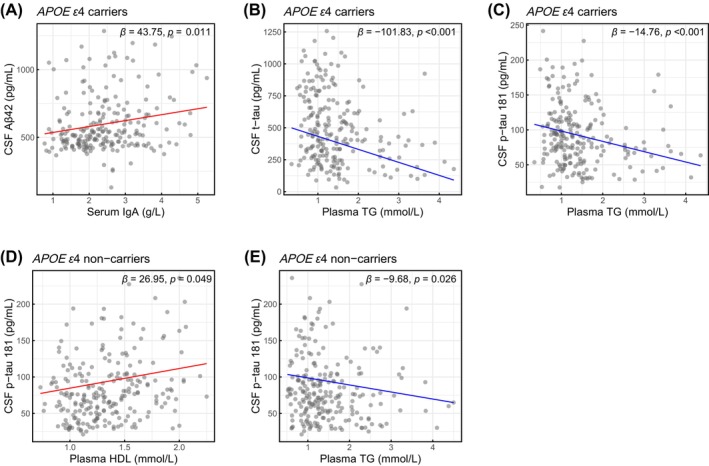
Associations between blood‐derived molecules and CSF biomarkers in *APOE* subgroups of AD patients. Utilizing the quantile regression method (at 0.50 quantile), shown are the relationships between serum IgA and CSF Aβ42 levels in *APOE ε*4 carriers (A), between plasma TG and CSF t‐tau levels in *APOE ε*4 carriers (B), between plasma TG and CSF p‐tau 181 levels in *APOE ε*4 carriers (C), between plasma HDL and CSF p‐tau 181 levels in *APOE ε*4 non‐carriers (D), and between plasma TG and CSF p‐tau 181 levels in *APOE ε*4 non‐carriers (E). All models were adjusted for age, sex, education level, and disease duration. All *p* values were adjusted by Benjamini/Hochberg (B/H) method. Aβ, amyloid‐beta; AD, Alzheimer's disease; *APOE*, *apolipoprotein E*; CSF, cerebrospinal fluid; HDL, high‐density lipoprotein; IgA, Immunoglobulin A; p‐tau, phosphorylated tau; TG, triglycerides; t‐tau, total tau.

Differences of associations between the blood‐derived biomolecules with CSF biomarkers in matched *APOE* subgroups were further validated by quantile regression analysis (at 50 quantile) and adjusted for possible confounding factors, including age, sex, education level, and disease duration (Figures 5 and S1). In the *APOE ε*4+ AD group, the level of serum IgA significantly and positively predicted CSF Aβ42 level (*β* = 43.75, B/H adjusted *p* = 0.011), the level of plasma TG significantly and negatively predicted CSF t‐tau level (*β* = −101.83, B/H adjusted *p* < 0.001) and CSF p‐tau 181 level (*β* = −14.76, B/H adjusted *p* < 0.001) (Figure 5A–C). In the *APOE ε*4− AD group, the level of plasma HDL significantly and positively predicted CSF p‐tau 181 level (*β* = 26.95, B/H adjusted *p* = 0.049), and the levels of plasma TG significantly and negatively predicted CSF p‐tau 181 level (*β* = −9.68, B/H adjusted *p* = 0.026) (Figure 5D,E). In total AD patients, the levels of plasma TG significantly and negatively predicted CSF t‐tau level (*β* = −62.68, B/H adjusted *p* = 0.006) and CSF p‐tau 181 level (*β* = −9.06, B/H adjusted *p* = 0.001). The level of plasma HDL significantly and negatively predicted CSF p‐tau 181 level (*β* = 21.94, B/H adjusted *p* = 0.008) (Figure S1).

## 4 Discussion

Polymorphism in the *APOE* gene is recognized as a major genetic risk factor for late‐onset AD (LOAD), with the *APOE ε*4 allele conferring an increased risk [4, 5]. However, the exact mechanisms by which *APOE ε*4 contributes to AD pathology are intricate and not completely understood. Moreover, further investigation is required to elucidate the relationships between *APOE ε*4, CSF biomarkers, and blood‐derived molecules. In this study, *APOE ε*4 carriers in AD patients exhibited higher levels of CSF t‐tau and p‐tau 181, indicating a more progressive type of disease than *APOE ε*4 non‐carriers. In the correlation analysis, the blood‐derived biomolecules associated with CSF biomarkers differed between *APOE ε*4 carriers and non‐carriers among AD patients, suggesting distinct roles of *APOE* genotypes in AD pathology.

Our findings revealed variations in fluid biomarker levels from the two *APOE* subgroups, which indicated the different roles of *APOE* genotypes in the progression of Alzheimer's disease. In AD participants, the increased levels of t‐tau and p‐tau 181 in CSF from *APOE ε*4 carriers aligned with previous studies, which suggested that *APOE ε*4 may be associated with tau pathology and thus leading to more progressive type of AD [21, 22]. We further conducted correlation and regression analyses to reveal the potential mechanisms by which *APOE ε*4 may influence AD pathology.

Cholesterol plays an indispensable role in the central nervous system, serving as a crucial component essential for axonal growth, synaptogenesis, and synaptic plasticity, which are fundamental to learning, memory formation, and neuronal repair [23]. Up until now, the evidence for the involvement of lipids and lipoproteins in the development of dementia remains inconclusive. Studies have reported that dyslipidemia is supposed to be one of the risk factors of AD [24, 25]. However, the relationships between plasma LDL, HDL, and AD remain controversial [26, 27, 28, 29, 30, 31, 32, 33]. In our study, the elevated plasma LDL and HDL levels in AD patients were consistent with previous findings [26, 27, 30, 33], but not supported by some other studies [28, 29, 31, 32]. Aligned with the previous study which found that a higher level of TG may be a protective factor of dementia development [34], the negative correlation between TG and CSF t‐tau and p‐tau 181 in *APOE ε*4+ AD subgroup indicated that TG may delay the AD progression by protecting neurons from damage and degeneration. According to previous studies, *APOE* may influence the risk of AD by disrupting the homeostasis of lipid metabolism [35, 36]. Our results showed that the correlations of HDL, LDL, and TG with the CSF biomarkers were differed between the *APOE ε*4 subgroups, which suggest that *APOE ε*4 may play a role in the lipid metabolism by interfering with different lipids in AD patients, thus further influence the AD pathology.

Neuro‐inflammation has been proved to be one of the vital mechanisms involved in the progression of AD [37]. As the most abundant complement protein, complement C3 is a reactant during the acute phase and may serve as an indicator of inflammation [38]. A plethora of research data showed that decreased levels of complement C3 may result in impaired immunological responses, consequently leading to reduced clearance of plaques in the AD‐prone brain, thus offering a plausible explanation of the phenomenon [11, 39, 40]. In the correlation analyses, serum complement C3 level was negatively related to CSF t‐tau protein in *APOE ε*4+ AD group, which may be supported by a proposed theory suggesting that *APOE ε*4 may have effects on the involvement of complement C3 in the amplification of pathological processes in the AD brain [11]. Bonham et al. also reported a conceptual model of the AD pathogenic cascade where a synergistic relationship between complement C3 and *APOE ε*4 results in advanced Alzheimer's associated pathology [41]. IgA is the most prominent immunoglobulin isotype found on mucosal surfaces, such as saliva, tears, and respiratory secretions [42]. According to the results reported by Pocevičiūtė et al. [10], the normal IgA response to AD‐related inflammatory events may be disturbed in *APOE ε*4 carriers. In the correlation and regression analyses, the positive relationship between IgA and CSF Aβ42 was significant only in *APOE ε*4+ AD group, which indicates that *APOE ε*4 may play a role in the inflammatory response related to IgA in AD patients. Further investigations are warranted to elucidate the precise mechanism underlying the influence of *APOE ε*4 on neuro‐inflammation in AD pathology.

Recognized as a systemic antioxidant, UA is believed to exert protective effects against neurodegeneration by scavenging reactive oxygen species [43]. In our study, AD patients exhibited a lower level of plasma UA than CN participants, which was consistent with previous studies [13, 44]. In our study, plasma UA was positively and significantly correlated with CSF t‐tau among *APOE ε*4+ AD patients in Spearman's correlation analysis, while this relationship was not validated by quantile regression analysis. These findings indicate that the effect of *APOE ε*4 on oxidative stress in AD pathology may be interfered by factors like age and sex, which still warrant further examination and validation.

Up to now, the diagnosis of AD primarily relies on the quantitative assessment of Aβ and tau protein levels in the CSF, or the utilization of neuroimaging modalities such as amyloid and tau protein positron emission tomography (PET), both of which are medically invasive and costly [45, 46]. However, recent investigations have underscored the relevance of early alterations in peripheral blood signatures in the onset of AD, and the role of blood biomarkers in the diagnosis of AD has been receiving more attention [47, 48]. In our study, the levels of plasma HDL, LDL, TG, and serum complement C3 were associated with levels of CSF biomarkers of AD. These assumptions suggested that blood‐derived biomolecules obtain the potential to be novel biomarkers to indicate the progression of AD. Our findings may illuminate future progress in AD pathology and the discovery of candidate blood biomarkers of AD, thus providing a foundation for early detection and therapeutic interventions of AD.

The limitations of this study need to be acknowledged. This study was conducted at a single study center of the hospital and had a limited sample size. This circumstance may introduce inherent selection biases, potentially affecting the reliability and generalizability of the results. Moreover, some of the data used for analysis are self‐reported by the patients (including education level, history of drinking, smoking, hypertension, statin therapy, and diabetes mellitus), which may introduce recall bias into the study. Another limitation of our study is its observational nature, which prevents us from determining a causative relationship. Further longitudinal studies with larger sample size will be necessary to confirm the associations between *APOE ε*4 and fluid biomarkers of AD, as well as to explore the underlying mechanism of *APOE ε*4 in AD pathology.

## 5 Conclusions

Our study revealed that *APOE ε*4 was associated with accelerated progression of AD pathology, manifesting as increased t‐tau and p‐tau 181 levels in CSF of AD patients. According to the correlation analysis between blood‐derived molecules and CSF biomarkers, *APOE ε*4 may contribute to the pathogenesis of AD by disrupting lipid metabolism and neuroinflammation. These findings suggested that *APOE ε*4 may play a crucial role in the pathogenesis of AD, and provided valuable insights for the exploration of novel biomarkers and the development of individualized therapeutic approaches.

## Author Contributions

W.W. designed and supervised the study. P.Z., Q.W., and D.G. recruited patients. J.J. collected samples for COAST dataset in this study. B.Z. and W.W. performed the statistical analysis. M.Q. wrote the first draft of the manuscript. All authors contributed to the interpretation of data and revision of the manuscript. W.W. and M.Q. obtained funding for the study.

## Conflicts of Interest

The authors declare no conflicts of interest.

## Supporting information


Appendix S1.

## Data Availability

Both raw and processed data of the COAST dataset that support the findings of the current study will be made available upon request to the corresponding author and the COAST committee to ensure that the privacy of the participants is protected. All analyses were conducted in R, version 4.3.2. Code used to generate the results presented in the manuscript are available from the corresponding author upon request.
