# Supplementary material for: The Effect of APOE ε4 on Alzheimer's Disease Fluid Biomarkers: A Cross‐Sectional Study Based on the COAST
Source: CNS Neurosci Ther. 2025 Jan 3;31(1):e70202. doi: 10.1111/cns.70202 (PMC11696244; doi:10.1111/cns.70202)
Supplement: Supplementary file 1 — Appendix S1. [file CNS-31-e70202-s001.docx]

**Supplementary Appendix**

**The effect of *APOE ε*4 on Alzheimer's disease fluid biomarkers: a cross-sectional study based on the COAST**

**Table of Contents**

[FIGURE S1. Associations between blood-derived molecules and CSF biomarkers in all matched AD patients. 1](#_Toc177246417)

[Table S1. Missing data and outliers for individuals enrolled in the study. 2](#_Toc177246418)

[Table S2. Spearman's correlation analysis of blood-derived biomolecules and CSF biomarkers in all matched AD patients. 4](#_Toc177246419)

[Table S3. Spearman's correlation analysis of blood-derived biomolecules and CSF biomarkers in matched *APOE ε*4 subgroups of AD patients. 5](#_Toc177246420)


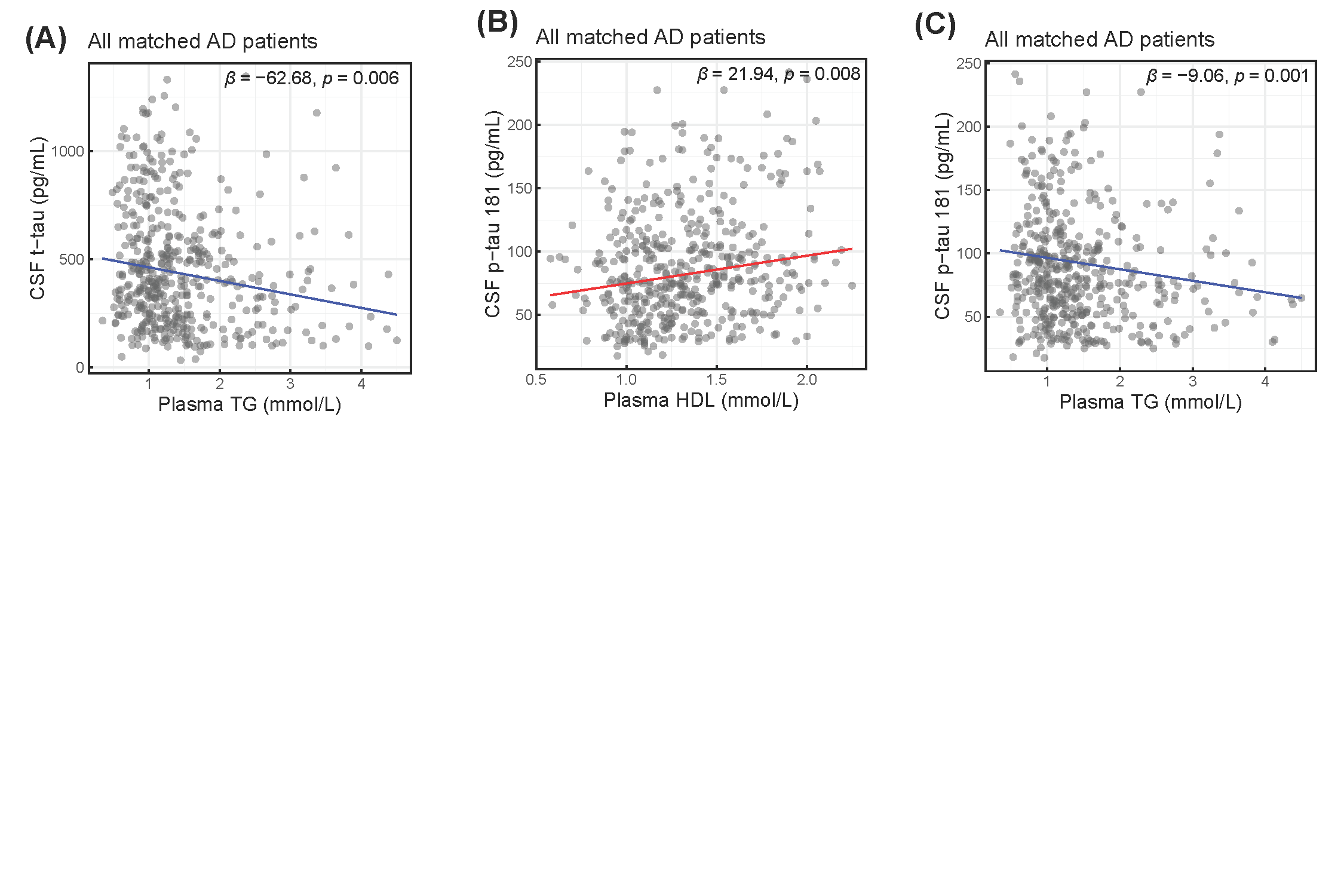


**FIGURE S1. Associations between blood-derived molecules and CSF biomarkers in all matched AD patients.**

Note: Utilizing the quantile regression method (at 0.50 quantile), shown are the relationships between plasma TG and CSF t-tau levels (A), between plasma HDL and CSF p-tau 181 levels (B), and between plasma TG and CSF p-tau 181 levels (C) in all matched AD patients. All models were adjusted for age, sex, education level, and disease duration. All *p* values were adjusted by Benjamini/Hochberg (B/H) method.

Abbreviations: AD, Alzheimer's disease; CSF, cerebrospinal fluid; HDL, high-density lipoprotein; p-tau, phosphorylated tau; t-tau, total tau; TG, triglycerides.

**Table S1. Missing data and outliers for individuals enrolled in the study.**

| **Variables** | **CN participants (*n* = 112)** | | |  | **AD patients (*n* = 575)** | | |  | **Non-ADD patients (*n* = 131)** | | |
| --- | --- | --- | --- | --- | --- | --- | --- | --- | --- | --- | --- |
|  | **Overall,**  ***n* (%)** | **Missing,**  ***n* (%)** | **Outliers,**  ***n* (%)** |  | **Overall,**  ***n* (%)** | **Missing,**  ***n* (%)** | **Outliers,**  ***n* (%)** |  | **Overall,**  ***n* (%)** | **Missing,**  ***n* (%)** | **Outliers,**  ***n* (%)** |
| AOO | – | – | – |  | 1 (0.17) | 1 (0.17) |  |  | – | – | – |
| Disease duration of AD | – | – | – |  | 1 (0.17) | 1 (0.17) | – |  | – | – | – |
| Educational level | 6 (5.36) | 6 (5.36) | – |  | 37 (6.43) | 37 (6.43) | – |  | 19 (14.50) | 19 (14.50) | – |
| BMI | 0 (0.00) | 0 (0.00) | – |  | 6 (1.04) | 6 (1.04) | – |  | 2 (1.53) | 2 (1.53) | – |
| Smoking | 0 (0.00) | 0 (0.00) | – |  | 0 (0.00) | 0 (0.00) | – |  | 0 (0.00) | 0 (0.00) | – |
| Drinking | 0 (0.00) | 0 (0.00) | – |  | 0 (0.00) | 0 (0.00) | – |  | 0 (0.00) | 0 (0.00) | – |
| Hypertension | 0 (0.00) | 0 (0.00) | – |  | 2 (0.35) | 2 (0.35) | – |  | 0 (0.00) | 0 (0.00) | – |
| Diabetes mellitus | 0 (0.00) | 0 (0.00) | – |  | 3 (0.52) | 3 (0.52) | – |  | 0 (0.00) | 0 (0.00) | – |
| Hyperlipidemia | 0 (0.00) | 0 (0.00) | – |  | 0 (0.00) | 0 (0.00) | – |  | 46 (35.11) | 46 (35.11) | – |
| MMSE | 6 (5.36) | 6 (5.36) | – |  | 5 (0.87) | 5 (0.87) | – |  | 6 (4.58) | 6 (4.58) | – |
| MoCA | 6 (5.36) | 6 (5.36) | – |  | 11 (1.91) | 11 (1.91) | – |  | 6 (4.58) | 6 (4.58) | – |
| CSF Aβ42 | 1 (0.89) | 0 (0.00) | 1 (0.89) |  | 15 (2.61) | 2 (0.35) | 13 (2.26) |  | 0 (0.00) | 0 (0.00) | 0 (0.00) |
| CSF Aβ40 | 2 (1.79) | 0 (0.00) | 2 (1.79) |  | 10 (1.74) | 2 (0.35) | 8 (1.39) |  | 3 (2.29) | 0 (0.00) | 3 (2.29) |
| CSF t-tau | 2 (1.79) | 0 (0.00) | 2 (1.79) |  | 7 (1.22) | 2 (0.35) | 5 (0.87) |  | 2 (1.53) | 0 (0.00) | 2 (1.53) |
| CSF p-tau 181 | 3 (2.68) | 0 (0.00) | 3 (2.68) |  | 11 (1.91) | 2 (0.35) | 9 (1.57) |  | 4 (3.05) | 0 (0.00) | 4 (3.05) |
| Plasma HDL | 2 (1.79) | 0 (0.00) | 2 (1.79) |  | 9 (1.57) | 4 (0.70) | 5 (0.87) |  | 3 (2.29) | 1 (0.76) | 2 (1.53) |
| Plasma LDL | 0 (0.00) | 0 (0.00) | 0 (0.00) |  | 5 (0.87) | 3 (0.52) | 2 (0.35) |  | 1 (0.76) | 1 (0.76) | 0 (0.00) |
| Plasma TG | 1 (0.89) | 0 (0.00) | 1 (0.89) |  | 7 (1.22) | 4 (0.70) | 3 (0.52) |  | 2 (1.53) | 1 (0.76) | 1 (0.76) |
| Plasma UA | 3 (2.68) | 0 (0.00) | 3 (2.68) |  | 5 (0.87) | 3 (0.52) | 2 (0.35) |  | 2 (1.53) | 1 (0.76) | 1 (0.76) |
| Serum IgA | 4 (3.57) | 3 (2.68) | 1 (0.89) |  | 17 (2.96) | 9 (1.57) | 8 (1.39) |  | 2 (1.53) | 1 (0.76) | 1 (0.76) |
| Serum C3 | 3 (2.68) | 3 (2.68) | 0 (0.00) |  | 13 (2.26) | 10 (1.74) | 3 (0.52) |  | 2 (1.53) | 1 (0.76) | 1 (0.76) |

*Note:* Outliers of each variable were defined as with z-scores more than three in each group (AD or CN). The variables of which the outliers were deleted included blood-derived biomolecules and CSF biomarkers.

Abbreviations: Aβ, amyloid-beta; AD, Alzheimer's disease; AOO, age of onset; BMI, body mass index; CN, cognitively normal; CSF, cerebrospinal fluid; C3, complement 3; HDL, high-density lipoprotein; IgA, Immunoglobulin A; LDL, low-density lipoprotein; MMSE, Mini-mental State Examination; MoCA, Montreal Cognitive Assessment; non-ADD, non-AD dementia; p-tau, phosphorylated tau; t-tau, total tau; TG, triglycerides; UA, uric acid.

**Table S2. Spearman's correlation analysis of blood-derived biomolecules and CSF biomarkers in all matched AD patients.**

| **Independent variables** | **Dependent variables** | ***n*** | ***r*** | **Adjusted *p*-value** |
| --- | --- | --- | --- | --- |
| Plasma LDL | CSF Aβ42 | 479 | -0.109 | 0.013^*^ |
| Serum IgA | CSF Aβ42 | 473 | 0.038 | 0.459 |
| Plasma HDL | CSF t-tau | 483 | 0.169 | 0.003^**^ |
| Plasma TG | CSF t-tau | 483 | -0.184 | 0.002^**^ |
| Plasma UA | CSF t-tau | 485 | -0.152 | 0.009^**^ |
| Serum C3 | CSF t-tau | 481 | -0.116 | 0.033^*^ |
| Plasma HDL | CSF p-tau 181 | 480 | 0.169 | 0.003^**^ |
| Plasma LDL | CSF p-tau 181 | 481 | 0.143 | 0.013^*^ |
| Plasma TG | CSF p-tau 181 | 480 | -0.172 | 0.003^**^ |
| Serum C3 | CSF p-tau 181 | 477 | -0.130 | 0.029^*^ |

*Note*: The fifth column lists the adjusted *p*-values according to Benjamini/Hochberg (B/H) method.

Abbreviations: Aβ, amyloid-beta; AD, Alzheimer's disease; CSF, cerebrospinal fluid; C3, complement C3; HDL, high-density lipoprotein; IgA, Immunoglobulin A; LDL, low-density lipoprotein; p-tau, phosphorylated tau; TG, triglycerides; t-tau, total tau; UA, uric acid.

^*^B/H adjusted *p* < 0.05, ^**^B/H adjusted *p* < 0.01.

**Table S3. Spearman's correlation analysis of blood-derived biomolecules and CSF biomarkers in matched *APOE ε*4 subgroups of AD patients.**

| **Independent variables** | **Dependent variables** | ***APOE ε*4+ AD patients** | | |  | ***APOE ε*4− AD patients** | | |
| --- | --- | --- | --- | --- | --- | --- | --- | --- |
|  |  | ***n*** | ***r*** | **Adjusted *p*-value** |  | ***n*** | ***r*** | **Adjusted *p*-value** |
| Plasma LDL | CSF Aβ42 | 243 | -0.163 | 0.042^*^ |  | 236 | -0.117 | 0.164 |
| Serum IgA | CSF Aβ42 | 237 | 0.211 | 0.030^*^ |  | 236 | -0.130 | 0.180 |
| Plasma HDL | CSF t-tau | 238 | 0.193 | 0.016^*^ |  | 245 | 0.158 | 0.104 |
| Plasma TG | CSF t-tau | 239 | -0.235 | 0.007^**^ |  | 244 | -0.142 | 0.104 |
| Plasma UA | CSF t-tau | 241 | -0.190 | 0.016^*^ |  | 244 | -0.129 | 0.120 |
| Serum C3 | CSF t-tau | 238 | -0.182 | 0.033^*^ |  | 243 | -0.041 | 0.626 |
| Plasma HDL | CSF p-tau 181 | 238 | 0.108 | 0.132 |  | 242 | 0.231 | 0.008^**^ |
| Plasma LDL | CSF p-tau 181 | 240 | 0.120 | 0.104 |  | 241 | 0.141 | 0.104 |
| Plasma TG | CSF p-tau 181 | 239 | -0.195 | 0.016^*^ |  | 241 | -0.143 | 0.104 |
| Serum C3 | CSF p-tau 181 | 237 | -0.129 | 0.127 |  | 240 | -0.111 | 0.174 |

*Note*: The fifth and eighth columns list the adjusted *p*-values according to Benjamini/Hochberg (B/H) method.

Abbreviations: Aβ, amyloid-beta; AD, Alzheimer's disease; *APOE*, *apolipoprotein E*; CSF, cerebrospinal fluid; C3, complement C3; HDL, high-density lipoprotein; IgA, Immunoglobulin A; LDL, low-density lipoprotein; p-tau, phosphorylated tau; TG, triglycerides; t-tau, total tau; UA, uric acid.

^*^B/H adjusted *p* < 0.05, ^**^B/H adjusted *p* < 0.01.
